# Supplementary material for: Heat*seq: an interactive web tool for high-throughput sequencing experiment comparison with public data
Source: Bioinformatics. 2016 Jul 4;32(21):3354–6. doi: 10.1093/bioinformatics/btw407 (PMC5079476; doi:10.1093/bioinformatics/btw407)
Supplement: Supplementary Data [file supp_32_21_3354__index.html]

Heat\*seq: an interactive web tool for high-throughput sequencing experiment comparison with public data — Heat\*seq: an interactive web tool for high-throughput sequencing experiment comparison with public data — Supplementary Data 

# Heat\*seq: an interactive web tool for high-throughput sequencing experiment comparison with public data

## Supplementary Data

files

- Supplementary Data - xlsx file
